# Double-pulse speckle contrast correlations with near Fourier transform limited free-electron laser light using hard X-ray split-and-delay

**DOI:** 10.1038/s41598-020-61926-y

**Published:** 2020-03-19

**Authors:** Wojciech Roseker, Sooheyong Lee, Michael Walther, Felix Lehmkühler, Birgit Hankiewicz, Rustam Rysov, Stephan O. Hruszkewycz, G. Brian Stephenson, Mark Sutton, Paul H. Fuoss, Marcin Sikorski, Aymeric Robert, Sanghoon Song, Gerhard Grübel

**Affiliations:** 1 Deutsches Elektronen-Synchrotron DESY, Notkestr. 85, 22607 Hamburg, Germany; 2 Frontier in Extreme Physics, Korea Research Institute of Standards and Science, Daejeon, 305-340 Republic of Korea; 3 Department of Nanoscience, University of Science and Technology, Daejeon, 305-350 Korea; 4 The Hamburg Centre for Ultrafast Imaging, Luruper Chaussee 149, 22761 Hamburg, Germany; 5 Materials Science Division, Argonne National Laboratory, Argonne, IL 60439 USA; 6 Department of Physics, McGill University, Montreal, Quebec H3A2T8 Canada; 7 Linac Coherent Light Source, SLAC National Accelerator Laboratory, Menlo Park, CA 94025 USA; 8 Present Address: Institute of Physical Chemistry, University of Hamburg, Grindelallee 117, 20146 Hamburg, Germany; 9 Present Address: European X-Ray Free-Electron Laser Facility, Holzkoppel 4, 22869 Schenefeld, Germany

**Keywords:** Techniques and instrumentation, X-rays

## Abstract

The ability to deliver two coherent X-ray pulses with precise time-delays ranging from a few femtoseconds to nanoseconds enables critical capabilities of probing ultra-fast phenomena in condensed matter systems at X-ray free electron laser (FEL) sources. Recent progress made in the hard X-ray split-and-delay optics developments now brings a very promising prospect for resolving atomic-scale motions that were not accessible by previous time-resolved techniques. Here, we report on characterizing the spatial and temporal coherence properties of the hard X-ray FEL beam after propagating through split-and-delay optics. Speckle contrast analysis of small-angle scattering measurements from nanoparticles reveals well-preserved transverse coherence of the beam. Measuring intensity fluctuations from successive X-ray pulses also reveals that only single or double temporal modes remain in the transmitted beam, corresponding to nearly Fourier transform limited pulses.

## Introduction

X-ray Free Electron Lasers (XFEL) based on Self Amplified Spontaneous Emission (SASE) deliver ultra-fast and spatially highly coherent hard X-ray radiation with extreme peak brightness (~10^12^ photons in a single pulse) making them ideal tools for studying atomic-scale dynamics in various condensed matter systems. The Linac Coherent Light Source (USA) was the first FEL to demonstrate lasing in the hard X - ray regime^1^ followed by SACLA (Japan)^2^, PAL-FEL (South Korea)^3^, European XFEL (Germany)^4^ and SwissFEL^5^. The most prominent time-resolved techniques used at the storage rings such as optical laser pump and X-ray probe^6–9^ methods or X-ray photon correlation spectroscopy (XPCS)^10^ have in the meantime also been recently demonstrated at the FEL sources^11–14^. The pump-probe approach has benefitted greatly from using femtosecond X-ray pulse duration provided at FEL facilities complemented by state of the art timing synchronisation schemes between optical laser and X-ray pulses^15,16^. These capabilities have enabled elaborate pump-probe^17–19^ and single-shot coherent imaging experiments^20,21^. However, replicating XPCS experiments at FELs is much more challenging because the intrinsic time-structure of the FEL sources is unsuitable for studying high-speed dynamical processes in many materials. Currently, most of FELs generate discrete bursts of X-ray radiation at a repetition rate ranging typically between 20 to 120 Hz. Exceptionally, European XFEL provides time structure of single pulses separated by 222 ns arranged into bunch trains arriving with the rate of 10 Hz^22^.

In conventional XPCS measurements, a disordered sample (e.g., colloidal suspension) is illuminated coherently by an X-ray beam and a grainy interference patterns (commonly referred as a “speckle” pattern^23^) is observed in the far field. The speckle pattern is related to the spatial arrangements of scatterers in the sample. If structural arrangements in the sample change with time, the corresponding speckle pattern will evolve accordingly. Thus, the dynamics of the sample can be traced by measuring temporal intensity fluctuations in the patterns by calculating an autocorrelation function in time-domain^10^ as follows

1 $${g}_{2}({\bf{q}},\tau )=\left\langle I({\bf{q}},t)I({\bf{q}},t+\tau )\right\rangle /{\left\langle I({\bf{q}},t)\right\rangle }^{2},$$

where I(**q**,t) is the intensity at the time *t* and at fixed length-scale (or wave-vector transfer **q**). The magnitude of the scattering vector is given by $| {\bf{q}}| =\left(4\pi /\lambda \right)\sin \theta$, where 2*θ* and *λ* are the scattering angle and wavelength, respectively.

**Figure 1:**
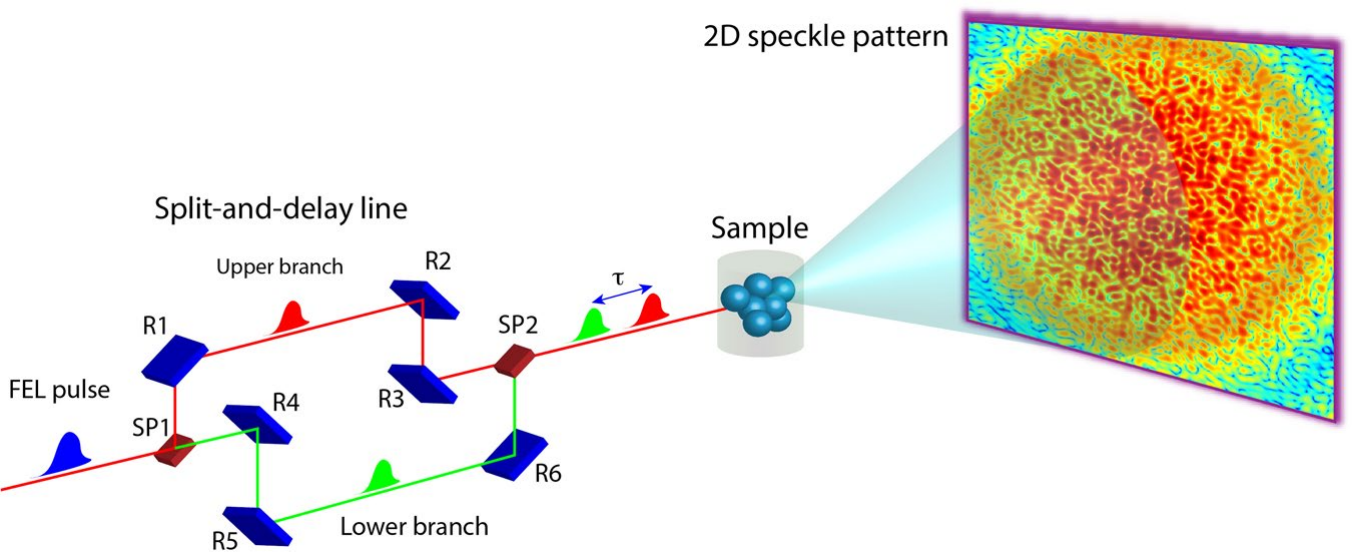
Illustration of the split-and-delay concept with the X-ray Photon Correlation Spectroscopy technique. A single LCLS X-ray pulse is split into two equal intensity pulses using the hard X-ray split-and-delay unit. The pulse paths are recombined and propagate collinearly in the sample direction. The summed speckle pattern from the two pulses is collected by a 2D detector. Contrast of the speckle pattern is analysed as a function of delay time *τ*.

In case of conventional XPCS (typically performed at 3rd generation storage rings) the lag time *τ* in equation (1) is given by the frame rate of the 2D detector. At the existing FELs, the time resolution of the XPCS measurements, in so called ‘sequential mode’^24^, is limited to the repetition rate of the FEL source. Furthermore, significant fluctuation in intensity and position of the FEL beam^25,26^, can hinder obtaining proper photon correlation between successive scattering signals and consequently can affect resulting autocorrelation function^11,12^. In principle, all these hindrances can be overcome by employing the “split-and-delay” approach^24^. As shown in Fig. 1, two split probing pulses are delayed with respect to each other before impinging on the sample. The scattering pattern is a sum of two independent coherent diffraction patterns. If the dynamics in the sample are sufficiently slow as compared to the time-delay between two pulses, the contrast, which is a measure of intensity fluctuations in the speckle pattern, will be equivalent to that of a static sample. However, when the characteristic time-scale is comparable or shorter than the time-delay, the speckle contrast will decrease in a known manner. Ultimately, the sample dynamics can be directly measured by monitoring the reduction in the speckle contrast *β* as a function of the time-delay *τ* between two probing pulses^14,27,28^ according to

2 $$\beta (\tau ,{\bf{q}})={\beta }_{0}\frac{{r}_{{\rm{sp}}}^{2}+1+2{r}_{{\rm{sp}}}{\left|f\left(\tau ,{\bf{q}}\right)\right|}^{2}}{{r}_{{\rm{sp}}}^{2}+1+2{r}_{{\rm{sp}}}},$$

where *β*_0_ and *f*(*τ*, **q**) are the single FEL pulse contrast and the intermediate scattering function (ISF)^10^, respectively. The ISF contains all dynamic properties of the investigated sample. Factor *r*_sp_ denotes intensity ratio of the two probing pulses.

Integration of the split-pulse method at the FEL sources will bring several noticeable advantages. First, the time resolution of the measurement is now defined by the X-ray pulse duration and the time-delay precision instead of detector frame rate or source repetition rate. Secondly, since the two pulses arise from the same electron bunch an intrinsic temporal synchronization is provided to study ultra-fast dynamics. More recently, there have been active efforts to deliver double FEL pulses with femtosecond-level time-delays^29–31^. Longer but highly discrete double-pulse delays (multiples of 350 ps) have been reported as well^32,33^. On the other hand, opto-mechanical means of splitting and delaying FEL pulses and generating desirable time-delays bridges the time gap between the achievable time-delays of the aforementioned double-pulse generation techniques. In the soft X-ray regime, various delay lines have been commissioned and are in operation^34,35^. However, replicating the similar scheme at the hard X-ray FEL sources has been a non-trivial task for a long time due to the lack of proper opto-mechanical components to split and recombine an FEL X-ray pulse. The first hard X-ray split-and-delay device has utilized Bragg crystal optics in a fixed 90-degree-scattering geometry^36,37^. More split-and-delay devices have been developed and are planned to be operated at LCLS^38^, SACLA^39^ and European XFEL^22^. Recently compact split-and-delay units has been demonstrated^40,41^.

In previous studies temporal and spatial properties of single FEL pulses have been characterised in detail^26,42^. Such information was crucial for carrying out single-pulse coherence-based FEL experiments such as Fourier Transform Holography or Coherent Diffraction Imaging. However, speckle contrast correlation based measurements using split-and-delay optics require characterizing coherence properties of the double FEL-pulses impinging on the sample, which has not been fully demonstrated so far.

In this work, we present investigations of spatial and temporal properties of individual split and delayed LCLS pulses passing hard X-ray split-and-delay device by analyzing speckle contrasts from nanoparticles in both spatial- and time-domain. We demonstrate high speckle contrast and delivery of nearly Fourier transform limited FEL pulses. These are crucial performance criteria necessary to operate the split-and-delay devices for ultra-fast coherence based experiments at FELs.

## Results

### Hard X-ray split-and-delay line

Figure 1 shows a schematic of the hard X-ray split-and-delay concept. The incoming X-ray pulse is first split by a beam-splitter crystal SP1 into two pulses, that propagate along two unequal rectangular paths. The optical path for one of the split pulses is defined by the Bragg crystals, denoted subsequently R1, R2 and R3 (hereafter called “upper branch”). The other split pulse is guided via the Bragg crystals, R4, R5 and R6 (hereafter called “lower branch”). Both pulses are brought back on a common path at the beam-mixer position SP2 and propagate co-linearly in the sample direction. The initial performance of the split-and-delay was investigated at 3rd generation storage ring sources^36,37^ and then its operation was successfully verified in ultra-fast XPCS study of nanosecond equilibrium structural dynamics^14^.

### Temporal coherence

**Figure 2:**
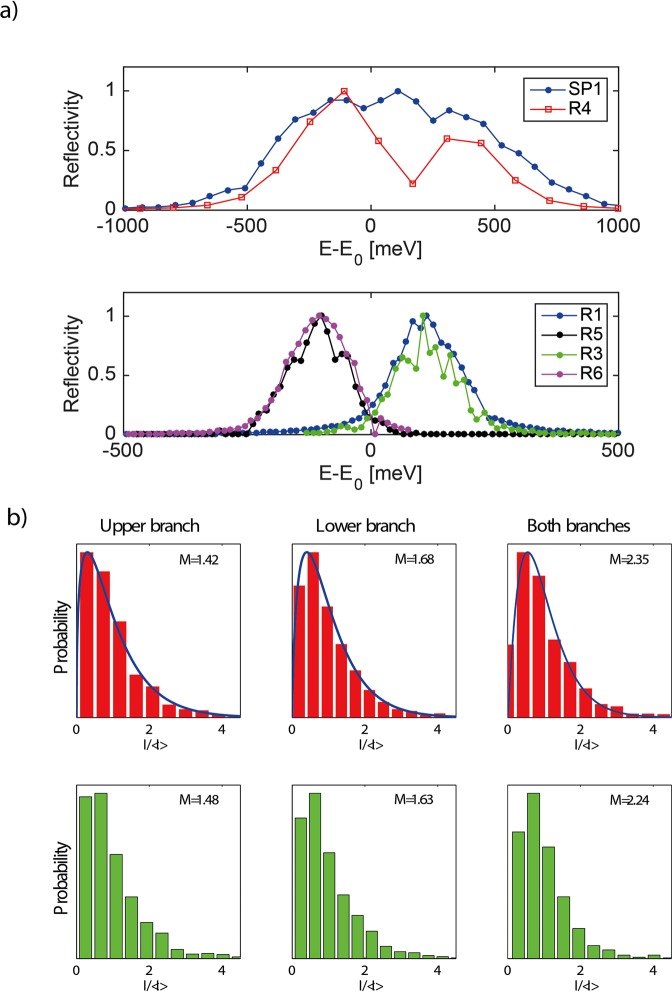
(**a**) Rocking curves of the Si(422) split-and-delay optics measured at an X-ray energy of *E*_0_ = 7.9 keV. (**b**) (Upper row) Histograms of the successive intensities and corresponding fits to Gamma distribution. (Lower row) Simulation results of the corresponding histograms.

Typically, a single SASE X-ray pulse consists of multiple temporal modes that manifest themselves in forms of sharp spikes in the frequency domain^43,44^. The width of the overall spectral profile is related to the pulse duration of the radiation while the characteristic width of the individual spike reflects the single longitudinal coherent mode. We expect that the split-and-delay device will impact the temporal coherence of the beam because the energy-bandwidth acceptance of the Si(422) Bragg optics is considerably narrower than the spectral width of the FEL radiation under normal operational conditions^1,25,45^. Figure 2(a) shows Bragg reflectivity scans of the split-and-delay optics, which define the effective energy band-pass of the X-ray beams after passing through the device. During the experiment, two slightly different X-ray energies were selected for the upper and the lower branches within the bandwidth that is provided by the large offset double crystal monochromator LODCM (Δ*E* = 980 meV)^46^. As shown in Fig. 2(a), the FEL radiation within the Bragg bandwidth of SP1 (ΔE = 116 meV) of the beam splitting crystal was reflected with high efficiency, which was confirmed by a dip in a rocking curve scan of the R4 crystal. The X-ray photons with energies outside the Bragg bandwidth were transmitted through SP1 and reflected by the R4, R5, R6 and SP2 crystals (see Fig. 1). The energy acceptance of the upper branch configuration is defined by the alignment of the R1, R2 and R3 Bragg reflectors. The resulting ΔE is 87 meV and 107 meV for upper and lower branch, respectively. The difference in ΔE of 20 meV between the branches indicates more accurate alignment of the lower brunch. The difference between the energies in the two branches is 230 meV. Such a small energy difference corresponds to a change of the scattering wave-vector q of 4 × 10^−6^ which is smaller than the Δq corresponding to the detector pixel size (Δq ≈ 1.8 × 10^−4^). Therefore slightly different energies should not affect the distribution of speckles in the two scattering patterns. The number of the temporal modes *M*_t_ that are contained within the effective energy bandwidths of the split-and-delay unit is evaluated by performing a statistical analysis on the fluctuation of the X-ray intensities *I* and fit by Gamma distribution^47^

3 $${P}_{\Gamma }(I)=\frac{{{M}_{{\rm{t}}}}^{{M}_{{\rm{t}}}}{I}^{{M}_{{\rm{t}}}-1}}{\Gamma \left({M}_{{\rm{t}}}\right){\left\langle I\right\rangle }^{{M}_{{\rm{t}}}}}\exp \left(-{M}_{{\rm{t}}}I/\left\langle I\right\rangle \right),$$

where Γ() is the gamma function. In our experiment, the relative intensities of individual X-ray pulses were obtained by summing up X-ray photons scattered from the static colloidal sample, which are measured by the 2D detector on a single pulse basis. However due to the electron energy jitter in the FEL machine, which effectively shifts the central frequency of the pink beam spectrum, it was necessary to bin the intensity data in accordance with the electron energies^25^.

Figure 2(b) (upper row) shows the intensity distributions of many successive FEL pulses that are transmitted through the upper, lower and both branches of the split-and-delay unit, respectively. The SASE FEL beam is transversely fully coherent. Therefore, the mode analysis in Fig. 2 shows only temporal modes. Our analysis yields the number of temporal modes to be $\left\langle {M}_{{\rm{t}}}\right\rangle$ = 1.42, 1.68 and 2.35, respectively. We note that, according to the Lorentzian approximation of the spectral transmittance^28^, coherence time ${\tau }_{c}=0.318\left(\lambda /c\right){\left(\Delta E/E\right)}^{-1}$, with *λ* denoting the wavelength and *c* the speed of light. We obtain *τ*_*c*_ of 15 fs and 12 fs for pulses passing the upper and the lower branch, respectively.

To support our findings a series of 1D FEL simulations^25^ are performed to model the intensity fluctuation after the delay line. The input parameters for the simulation such as electron energy, peak currents and bunch charge are derived from the actual operational parameters of LCLS during our experiments. The effects of monochromatization is calculated using the *DuMond* approach, where the Bragg diffraction profile is obtained from the overlap between the source divergence and the measured bandwidth of the upper and lower branch of the split-and-delay unit at a given wavelength. The simulation provides very consistent results with our experimental finding, in which the numbers of temporal modes are *M*_t_ = 1.48, *M*_t_ = 1.63 and *M*_t_ = 2.24 for the configurations of the upper, lower and both branches, respectively. The slight difference in the mode number between the lower and upper branches is due to different spectral transmittance of each branch. The energy bandwidth of the Si(422) Bragg crystal reflection^48^ (Δ*E*/*E* = 1.47 × 10^−5^) used in the split-and-delay unit closely matches the spectral width of a single temporal mode of SASE radiation under the nominal operational condition of LCLS^25^. In particular, when the spectral bandwidth of X-ray pulses matches or even overfills the bandwidth acceptance of the Si(422) reflection, the X-ray pulses after the split-and-delay unit becomes nearly Fourier transform limited (i.e. *τ*_p_ ≈ *τ*_c_) and thus enables the delivery of X-ray pulses with a single-temporal mode. One of the widely used criteria for characterizing light sources is the photon degeneracy parameter $\delta =\left\langle I\right\rangle$/*M* (i.e., number of photons in a single mode M), where *I* is the incoming photon number. For instance, the *δ* at incoherent light sources such as a storage rings is typically much less than 1. In our experiment, *δ* measured at the XCS instrument at LCLS after the insertion of the split-and-delay optics is 2 × 10^7^.

We also note that the narrower spectral bandwidth of the X-rays after the split-and-delay device effectively increases the coherence volume and thus allows speckle contrast measurement at higher scattering angles^25^, which is crucial condition for studying dynamics with atomic resolution^49^. On average, we expect that approximately two independent temporal modes are delivered to the sample. In this case, while the pulse duration of the X-ray pulse should remain mostly unchanged, we expect that the longitudinal coherence length will be slightly reduced. We note that the number of temporal modes is related to the average FEL pulse length $\left\langle {\tau }_{{\rm{p}}}\right\rangle$ of X-ray pulses via a relation^42^, $$\left\langle {\tau }_{{\rm{p}}}\right\rangle \ =\ $$M_t_ × *τ*_c_. Based on the coherence length given by the Bragg crystals and the number of modes, we measure the X-ray pulse duration of about 35 ± 5 fs (including effects of temporal broadening due to transient response of the crystal and geometrical dispersion^50^) after the split-and-delay optics. Slightly shorter pulse duration of 29 ± 14 fs was achieved without the split-and-delay line^42^.

### Spatial coherence

**Figure 3:**
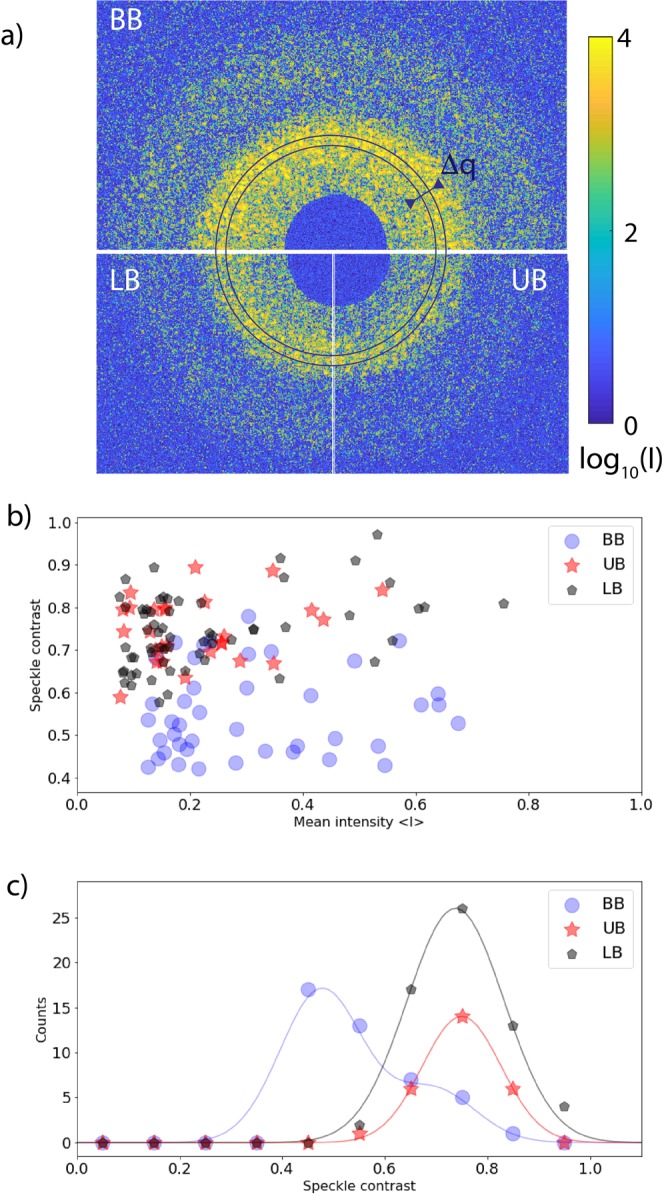
(**a**) Speckle patterns measured from the static sample with the split-and-delay unit configured in upper branch (UB), lower branch (LB) and both branches (BB). For these measurements, the delay was set to 20 ps. (**b**) Speckle contrast *β* as a function of the mean photon FEL pulse intensity taken with the upper, lower and both branches, respectively. (**c**) Observable contrast for upper, lower, both branches, respectively. Solid lines are Gaussian fits to corresponding speckle contrast distributions.

The transverse coherence properties of the LCLS beam passing through the split-and-delay optics were investigated by analyzing static speckle patterns of a dried PMMA colloidal sample. Since the speckle pattern from the static sample does not change in time, the contrast preservation of the split-and-delay unit can be investigated in great detail. Figure 3a shows single-pulse static speckle patterns with the split-and-delay device operated with upper branch (UB), lower branch (LB) and both branches (BB), respectively. We note that the single branch speckle patterns were recorded when either the upper or lower branch of the split-and-delay unit was blocked by a beamstop. Speckle contrast *β* at q = 3 × 10^−3^ Å^−1^ was evaluated from 150 successive scattering patterns as shown in Fig. 3(b). The contrast of recorded speckle patterns was analyzed with the help of the negative binomial probability distribution^28^,

4 $$P(I)=\frac{\Gamma (I+M)}{\Gamma (M)\Gamma (I+1)}{\left(1+\frac{M}{\left\langle I\right\rangle }\right)}^{-I}{\left(1+\frac{\left\langle I\right\rangle }{M}\right)}^{-M},$$

where *M* denotes number of speckle modes. The contrast *β* of a speckle pattern is related to *M* according to *β* = 1/*M*. For M = 1, equation (4) simplifies to

5 $$P(I)=\frac{1}{\left\langle I\right\rangle }\exp \left(-I/\left\langle I\right\rangle \right),$$

which reflects the intensity distribution of the fully developed speckle pattern and indicates the conditions of fully coherent illumination. The probability density function, *P*(*I*), was obtained by histogramming the intensities of selected **q** values. A fit procedure was applied to obtain values of *M*.

The mean speckle contrast values measured in the single branch configuration (i.e., *β*_UB_ = 0.76 ± 0.04 and *β*_LB_ = 0.78 ± 0.04) are equivalent within uncertainty. When both branches are in place, the speckle contrast decreases to *β*_BB_ = 0.57 ± 0.02. This reduction indicates that the X-ray beams from each branch are not fully overlapped at the sample position. We note that, during the data acquisition, the split-and-delay device was not equipped with inline beam position monitors that are capable of detecting individual FEL pulses. Since the two branches of the split-and-delay unit select out two independent parts from a SASE spectrum, we expect that the intensity in the two branches will vary on shot-to-shot basis. In the most extreme cases, only one branch delivers the photons to the sample per single SASE pulse. As a result, the contrast at a fixed delay time will vary independently of the sample dynamics. In order to distinguish between single and double shot illumination in the summed speckle patterns, we fit histograms Gaussian peaks as shown in Fig. 3(c). The probability of the contrast obtained with a single delay branch shows a single peak centered at 0.74 for the upper and lower branch. In the case of the two branch operation, the obtained probability of the contrast peaks at 0.48 and has a shoulder extending into higher contrast values. The peak value corresponds to double-pulse contrast while the the higher contrast of the shoulder reflects a mixture of double and single pulse illumination. Regardless, such high speckle contrasts (i.e., values above 0.5) obtained from using the full size of the X-ray beam demonstrate that the transverse coherence nature of the FEL beam is well preserved after the split-and-delay optics.

## Discussion

Most of the XPCS studies to date have probed relatively large length scale (≈100 nm) structures or dynamics occurring at slow time scales (≈ms). Such constraints in the selection of the sample systems are mandated by lack of sufficient coherent photon flux and time resolution. Hard X-ray FEL sources provide sufficient photon flux and coherence to acquire single-shot speckle patterns from atomic scale ordering^42,50^, while the chaotic nature of the source (intensity and positional instabilities) hinders dynamical studies. The promise of developing the split-delay device has been to overcome such limitations and measure dynamics at atomic length and time scales. In this study, we have demonstrated the coherence properties of the individual FEL pulses after the hard X-ray split-and-delay unit by analysing static speckle patterns. Our result reveals that the spatial part of the coherence is well preserved and that only one or two temporal modes are transmitted improving the longitudinal coherence of the radiation. We also demonstrated its femtosecond time-delay capability and stability via a low coherence light interferometry^51^.

However, one significant challenge still remains - is there sufficient coherent X-ray flux remaining after the split-and-delay to study femtosecond dynamics at large **q** values ? At large scattering angles (high **q** values), the photon flux arriving at the detector drops dramatically. Furthermore, the detector must be placed sufficiently far away from the sample to resolve speckles. In our previous studies^42,50^, we measured a typical photon flux of 0.03 photons/pixel/pulse from gold nanopowder at q = 2.6 Å^−1^. Subsequent studies have shown that it is still possible to obtain meaningful speckle contrast from such low intensity speckle, as it was demonstrated for scattering signals from amorphous solids and liquids using coherent x-rays after Si(111) monochromator^49,52^. While the narrower energy bandwidth of Si(422) reflection used in the split-and-delay helps to improve the longitudinal coherence, it decreases the photon flux considerably. Here we evaluate the associated statistical uncertainties in *β* after the split-and-delay is inserted in the beam. At high **q** regimes, since the scattering signals mostly consist of 1 or 2 photon events, the signal-to-noise ratio (SNR) in our experimental conditions can be evaluated as follows^49^

6 $$SNR=\beta \left\langle I\right\rangle \sqrt{\frac{{n}_{{\rm{px}}}\times {n}_{{\rm{pt}}}}{2(1+\beta )}},$$

where *n*_px_ and *n*_pt_ are number of pixels on the 2D detector and number of speckle patterns measured in an experiment, respectively.

**Figure 4:**
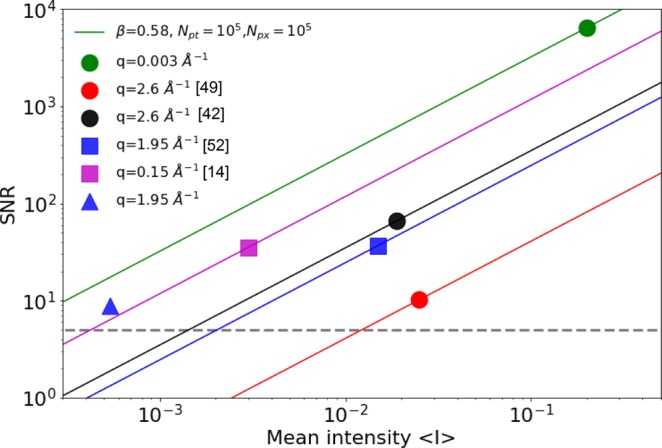
Signal-to-noise (SNR) ratio as a function of mean photon intensity measured for different experimental configurations at FEL during static speckle contrast (circles) and dynamic (squares) XPCS studies reported in^14,42,49,52^. Horizontal dashed line denotes the SNR of 5. Blue triangle shows calculated SNR for the ps - ns XPCS study with the split-and-delay on the water sample based on experimental conditions described in^52^.

Figure 4 shows how the SNR in the speckle contrast varies as a function of mean photon intensity for various experimental conditions (*β*,$\left\langle I\right\rangle$, *n*_px_, *n*_pt_) reported in high **q** static contrast measurements and XPCS studies at FEL^14,42,49,52^. The minimum signal-to-noise ratio required for XPCS is 5^53^ which corresponds to 20% uncertainty of measured contrast *β* (see horizontal dashed line in Fig. 4). For XPCS measurements at FELs, typically *n*_px_ > 10^4^ pixels on the detector were used to resolve *n*_pt_ > 10^4^ speckle images. The blue square in Fig. 4 shows the SNR from a water sample achieved in^52^. Extending these studies to ps-ns time scales,e.g., a critical time scale to measure ISF of liquid water^54^, can be achieved using the split-and-delay line. When the photon flux is reduced by a factor of 28 upon an insertion of the split-and-delay unit in the beam^55^, the expected SNR in the contrast *β* will proportionally decrease. However, thanks to high energy resolution provided by delay line optics the speckle contrast will increase at high scattering angles. For instance at q = 1.95 Å^−1^ the expected contrast is 2.2 times higher compared to Si(111) optics used in^52^. Blue triangle in Fig. 4 shows the signal-to-noise ratio of 8 expected for experimental conditions reported in^52^, *n*_pt_ = 10^6^ and the split-and-delay optics.

In conclusion, we successfully measured the spatial and spectral properties of individual split and delayed LCLS pulses by using the hard X-ray split-and-delay unit. A high hard X-ray photon degeneracy parameter presented in this work opens a new venue for performing ultra-fast photon correlation experiments as well as raising possibilities for pursuing quantum^56^ and nonlinear optical experiments^57^ in the X-ray regime. In particular in materials science, understanding complex diffusive and vibrational dynamics of atoms and molecules in an amorphous system, such as liquid and glasses remain a great challenge because such task requires a capability to measure ensemble-averaged (spatial and temporal) atomic movements occurring at sub-nanometer length and sub-nanosecond time scales. Until now, only sophisticated theoretical models^58^ or molecular level simulation^59^ have mostly provided viable means to study such phenomena. Finally, a combined use of the X-ray split-and-delay unit and FEL sources offers novel opportunities to directly observe the equilibrium dynamics of atomic motions in amorphous materials down to atomic length and time scale. Furthermore such a promising prospect of performing ultra-fast X-ray speckle correlation experiments are now further encouraged by the recent arrival of the self-seeded operation at the FEL sources.

## Methods

### Sample

The static sample was prepared by drying a suspension of Poly(methyl methacrylate) (PMMA) colloidal spheres inside 0.7 mm quartz capillary. The particles have a radius of 126 nm with a polydispersity of 7%.

### Experiment

The impact of the split-and-delay unit on the properties of FEL pulses has been investigated at the XCS instrument^46^ of the LCLS at SLAC National Accelerator Laboratory. Photon pulses delivered to the instrument were monochromatized to ΔE/E = 1.24 × 10^−4^ at E = 7.9 keV using (LODCM) before reaching the split-and-delay unit. The X-ray beam was focused at the sample position with beryllium refractive lenses. The split-and-delay unit was operating with Si(422) crystal optics. The scattered intensity was recorded by a direct-illumination CCD (Princeton Instrument, LCX). The LCX camera comprised 1340 × 1300 pixels, each of dimension 20 *μ*m × 20 *μ*m. The electronic noise of the CCD was accounted for in the analysis by measuring a series of single dark images without X-ray beam and its average was subtracted from each recorded data set. A beam stop was mounted in front of the detector to prevent it from being illuminated by the direct FEL beam. The pixels obstructed with the beam stop were masked and not used the analysis.

### FEL simulations

The spectral output of the LCLS beam after the delayline is obtained by performing 1-D FEL simulation near SASE saturation regime. The input parameters for the simulation such as electron energy, peak currents and bunch charge are derived from the actual operational parameters of LCLS during our experiments. The effects of monochromatization is calculated using the DuMond approach, where the Bragg diffraction profile is obtained from the overlap between the source divergence and the intrinsic bandwidth of the Bragg crystals of the delayline at a given wavelength. The simulation is repeated over 1000 iterations to ensure the statistical reliability.
